# The Story behind COVID-19: Animal Diseases at the Crossroads of Wildlife, Livestock and Human Health

**DOI:** 10.1017/err.2020.45

**Published:** 2020-04-27

**Authors:** Nicolas DE SADELEER, Jacques GODFROID

**Affiliations:** * Professor of Law, Jean Monnet Chair, University of Saint Louis, Brussels, Belgium; email: desadeleer.nicolas@gmail.com.; ** Professor of Microbiology, UiT – The Arctic University of Norway, Tromsø, Norway.

## Abstract

A number of virological, epidemiological and ethnographic arguments suggest that COVID-19 has a zoonotic origin. The pangolin, a species threatened with extinction due to poaching for both culinary purposes and traditional Chinese pharmacopoeia, is now suspected of being the “missing link” in the transmission to humans of a virus that probably originated in a species of bat. Our predation of wild fauna and the reduction in their habitats have thus ended up creating new interfaces that favour the transmission of pathogens (mainly viruses) to humans. Domesticated animals and wild fauna thus constitute a reservoir for almost 80% of emerging human diseases (SARS-CoV, MERS-CoV, Ebola). These diseases are all zoonotic in origin. As if out of a Chinese fairy tale, the bat and the pangolin have taught us a lesson: within an increasingly interdependent world, environmental crises will become ever more intertwined with health crises. Questions relating to public health will no longer be confined to the secrecy of the physician’s consulting room or the sanitised environment of the hospital. They are now being played out in the arena of international trade, ports and airports and distribution networks. Simply put, all human activity creates new interfaces that facilitate the transmission of pathogens from an animal reservoir to humans. This pluri-disciplinary article highlights that environmental changes, such as the reduction in habitats for wild fauna and the intemperate trade in fauna, are the biggest causes of the emergence of new diseases. Against this background, it reviews the different measures taken to control, eradicate and prevent the emergence of animal diseases in a globalised world.

## I. Introduction

Today, the world is facing the ongoing COVID-19 pandemic, caused by the SARS-CoV2 coronavirus. In February 2020 in China, an overall case–fatality rate of 2.3% was estimated for COVID-19. Every day, we are watching in real time the evolution of the pandemic around the world.^1^ In March 2020, the centre of the pandemic moved to Europe, then later shifting to the USA in April.

COVID-19 was first discovered in November 2019 in China, Hubei Province. This pandemic has largely crowded out international attention concerning another viral disease known as African swine fever (ASF), which is caused by an asfivirus and is harmless to humans. Currently, this epidemic viral disease is spreading around world in Suidae (animals such as pigs). ASF causes high mortality in domestic pigs – approaching 100% – while it may persistently infect its natural hosts (ie warthogs, bush pigs and soft ticks of the genus *Ornithodoros*) with no clinical signs. In particular, ASF is devastating China’s domestic pig population, which accounts for half of the world’s domestic pig population.^2^ For 18 months, the Chinese authorities and farmers have been battling ASF, with millions of pigs slaughtered within a few months.

The two crises to some extent overlap. The ongoing COVID-19 crisis has further exacerbated shortages of pork meat. Moreover, COVID-19 has significant economic impacts given that restrictions have been placed on the pork meat value chain. Importantly, besides direct transmission of ASF virus between pigs, transmission occurs through pork and pork products (raw/frozen/dried/undercooked), in which the virus can survive for a long time.

For both COVID-19 and ASF, there is no treatment and no vaccine.

Both COVID-19 and ASF crises remind us that new infectious diseases can emerge at any time in livestock and wildlife. Against this background, it must be considered that 80% of emerging diseases in humans are zoonoses (their origin is found in animals) and about 70% of these zoonoses originate from wild animals.

This opinion paper pursues three objectives. Firstly, it explains the origin as well as the significant socio-economic impacts of pathogens, principally viruses of zoonotic origin. Secondly, it highlights the importance of biodiversity, the reduction of wildlife habitats and enhanced predation on wildlife as drivers of disease emergence. Thirdly, it reviews the different measures taken to control, eradicate and prevent the emergence of animal diseases in a globalised world. The purpose of combating animal diseases is to protect people by protecting their livelihoods (ie livestock) and avoiding transmission of zoonotic agents (viruses, bacteria and parasites) at wildlife/livestock/human interfaces.

This article is in essence pluri-disciplinary as it encompasses scientific issues as well as legal considerations. Given the sheer complexity of the issues covered, prospective readers will be directed throughout the manuscript towards reliable sources of information in order to gain a greater insight into specific topics.

The article is structured as follows. Section II focuses on the worldwide crisis facing biodiversity, which compounds the interface between wild animals and human beings. Adopting a scientific perspective, Section III sheds light on disease transmission at the interface between wildlife, livestock and humans, whilst Section IV discusses the prevention and control of transmissible animal diseases, including zoonoses. From a legal perspective, Section V highlights the weakness of the regulatory approach in preventing and controlling transmissible animal diseases.

## II. Biodiversity in crisis

Although about 1,320,000 animal species have been identified, scientists estimate the total number of species on earth at more than 10 million. In addition, biodiversity is not distributed evenly across the planet. Generally speaking, on the continents, it increases from the poles towards the equator, whilst in the oceans the increase is much less pronounced, and sometimes operates in reverse.

Biodiversity is passing through a period of major crisis. In November 2017, 15,364 scientists from 184 countries signed a “Warning to Humanity” published in *BioScience* expressing their concerns about the future of wildlife.^3^ The signatories of this manifesto stressed that humanity is on a collision course with the natural world as ecosystems are being pushed beyond their capacities to support the web of life on this planet. In their wake-up call, they warned us that we are unleashing the sixth mass extinction,^4^ in which many forms of life are likely to disappear. With the rate of extinction running at more than 100,000 times the background rate, half of all the world’s species could become extinct within a few decades if humanity is unable to endorse “a more environmentally sustainable alternative to business as usual”.^5^

The nature and magnitude of the threats are well known, and they include fragmentation and habitat loss, over-harvesting of fauna, trading in species, etc. Despite past warnings, threats to biodiversity have continued unabated, making it impossible to meet the target of halting biodiversity loss in accordance with the Aichi biodiversity targets set by the UN Convention on Biological Diversity (hereafter CBD).^6^ As discussed as above, many of these threats compound the spread of viruses among humans.

The task of reducing these threats is daunting. Many of the conservation measures undertaken by State authorities have been carried out in accordance with the CBD. Under that Agreement, conservation and sustainable use are regarded as the cornerstones of biodiversity policy.^7^ Although conservation is not defined in the CBD, its preamble states that “the fundamental requirement for the conservation of biological diversity is the in-situ conservation of ecosystems and natural habitats and the maintenance and recovery of viable populations of species in their natural surroundings”. The CBD defines “sustainable use” as “the use of components of biological diversity in a way and at a rate that does not lead to the long-term decline of biological diversity, thereby maintaining its potential to meet the needs and aspirations of present and future generations”.^8^ The rationale of the latter concept is that it is possible to use biodiversity in a manner in which ecological processes, species and genetic variability remain above the thresholds needed for long-term viability.^9^

As the assessment of the status of biodiversity lies at the core of the CBD, it is important to determine whether biodiversity trends can differ and also what the causes are of these differences. It has recently been demonstrated that biodiversity may rise or fall depending on metrics or taxa. However, few monitoring programmes have the resources needed in order to measure changes to biodiversity components accurately in time and space. It must be considered that, unless greater resources are deployed, there is often a risk of incorrect diversity changes being extrapolated based on individual measurements, regions or emblematic taxonomic groups.^10^

While wildlife diseases alone may currently threaten few species, pathogens may be a significant threat to already-endangered species. This would be the case when diseases interact with other drivers such as habitat loss, climate change, overexploitation, invasive species and environmental pollution.^11^

## III. Animal diseases: the wildlife, livestock and human interface

### 1. Introductory remarks

Founded in 1924, the World Organisation for Animal Health (hereafter OIE, for Office International des Epizooties) is the intergovernmental organisation responsible for improving animal health worldwide.^12^ The goal of the founding parties was to put an end to the epidemic diseases (named epizootics) that were devastating their livestock. These objectives are still among the priority missions of the OIE, both for diseases that affect animals only and those also that are transmissible to humans (called zoonoses). For the latter type, collaboration agreements have been concluded with the World Health Organization (WHO) of the United Nations (UN).

The Food and Agricultural Organization (FAO)^13^ of the UN is frequently involved in emergency responses triggered by severe animal (infectious) diseases, which have the ability to spread rapidly over large geographical areas (“transboundary animal diseases”, or TADs) into previously unaffected countries or regions, or by natural and human-made disasters such as droughts, floods, earthquakes, etc. Activities such as early warning and surveillance of emerging diseases are carried out jointly with the OIE.

The parties to these two international organisations are required to report “important” livestock diseases. However, diseases affecting wildlife that are likely to harbour zoonotic pathogens are reported on a voluntary basis. This entails a risk that some “important” diseases may not be duly reported to the two international organisations.

In order to understand the control of animal diseases, we shall classify them below according to their respective epidemiology. Each epidemiological category will be illustrated with reference to specific cases. The aim of this section is to highlight that routes of transmission can involve different livestock (eg pigs) and different wildlife species (eg bats). In addition, transmission routes for the same virus are likely to differ over space and time. Accordingly, these transmission routes are fraught with uncertainty. As this article does not seek to be exhaustive, some diseases that have caught the public attention will be mentioned but not developed in detail.

### 2. Zoonotic diseases with pandemic potential

Avian influenza viruses (AIVs) are designated as highly pathogenic avian influenza (HPAI) or low-pathogenicity avian influenza (LPAI) based on the molecular characteristics of the virus and the ability of the virus to cause disease and mortality in chickens in a laboratory setting. AIVs rarely infect humans, although outbreaks of some AIVs in poultry have been associated with illness and death in humans.^14^ The most severe influenza pandemic, the so-called “Spanish Flu”, was caused by an AIV and is estimated to have caused 20–50 million deaths in 1918–1919.^15^ The first influenza pandemic of the twenty-first century occurred in 2009–2010 and was also caused by an AIV. Swine influenza (swine flu) is a respiratory disease in pigs caused by AIVs that regularly cause outbreaks of influenza in pigs, but usually cause few deaths. Sporadic human infections with swine flu have occurred. When this happens, these viruses are called “variant viruses”. As pigs are susceptible to avian, human and swine influenza viruses, they may potentially be infected with influenza viruses from different species (eg ducks and humans) at the same time. If this happens, it is possible for the genes of these viruses to mix and create a new virus. If this new virus causes illness in humans and can be transmitted easily from person to person, an influenza pandemic can occur. This is what most likely happened in North America when an AIV with swine, avian and human genes emerged in the spring of 2009.^16^ Following this pandemic, the WHO International Health Regulations Review Committee declared in 2011 that “the world is ill-prepared to respond to a severe influenza pandemic or to any similarly global, sustained and threatening public-health emergency”. The WHO therefore implemented the “Pandemic Influenza Preparedness (PIP) Framework”,^17^ a global approach to pandemic influenza preparedness and response.

The AIV that caused the 2009–2010 pandemic is now a regular human flu virus and continues to circulate seasonally throughout the world.

### 3. Pathogens infecting different livestock species

Foot-and-mouth disease (FMD), which is caused by a picornavirus, affects all cloven-hoofed animals, both domesticated and wild. FMD is characterised by fever and blister-like sores on the tongue and lips, in the mouth, on the teats and between the hooves. FMD is a TAD that severely affects the production of livestock and disrupts regional and international trade in animals and animal products. A very severe outbreak of FMD occurred in the UK during 2001, costing in excess of £3 billion, with 6.5 million animals slaughtered during control efforts. The first farm to suffer an outbreak was a pig farm, although the source of the virus remains a mystery.^18^ FMD is not zoonotic.

### 4. Wildlife as a reservoir of pathogens for livestock and/or humans

This is actually the case for ASF, FMD and AIVs. For ASF, whilst wild pigs in Africa do not show clinical signs, the infection is usually deadly for wild boars in Europe. Both African wild pigs and European wild boars can transmit the virus to domestic pigs. Pigs that are reared outdoors are particularly at risk. In South Africa, FMD is asymptomatic in buffalo roaming in Kruger National Park. The virus can be transmitted to domestic cattle that come into contact with infected buffalo when the western boundary fence of the park is not well maintained or is broken and no longer wildlife-proof.^19^ The reservoir of AIVs are waterfowl, mainly ducks (Anatidae). Several duck species are naturally resistant to AIVs. These duck species can shed and spread the virus (including HPAI, almost 100% lethal for poultry) from both the respiratory and intestinal tracts while showing few or no symptoms of disease. AIVs are maintained in waterfowl populations by water-borne transmission. Migratory birds are a reservoir of AIVs and are involved in the spread of virus diversity that has contributed to previous pandemic events.^20^

### 5. COVID-19, Nipah and Ebola virus outbreaks: zoonoses with or without an intermediate host?

The Nipah virus (NiV) outbreak in Malaysia (September 1998 to May 1999) resulted in 265 cases of acute encephalitis with 105 deaths and the near collapse of its billion-dollar pig-farming industry. Humans contracted the infection from close contact with infected pigs, and on this basis the pig culling that eventually stopped the outbreak was implemented. The virus was isolated from the urine and saliva of Malaysian Island fruit bats, suggesting that these bats were natural reservoir hosts of NiV. It is probable that initial transmission of NiV from bats to pigs occurred in late 1997/early 1998 through the contamination of pig swill by bat excretions. Fruiting failure of forest trees during the El Niño-related drought and fires caused by people in Indonesia in 1997–1998 resulted in the migration of these forest fruit bats to pig farms.^21^ In 2001, during NiV outbreaks in Bangladesh, it was shown that ingestion of date palm sap contaminated with saliva or excreta from infected fruit bats was the main spill-over route, with no involvement of pigs as intermediate hosts. During these outbreaks, person-to-person transmission occurred among persons in close contact, including healthcare workers.^22^ There is currently no available vaccine.

It is believed, but has not been demonstrated, that infection with the Ebola virus occurs initially through contact with an infected animal, such as fruit bats or via intermediate amplifying hosts, such as a non-human primate.^23^ After that, the virus spreads from person to person. The largest Ebola outbreak in history was first reported in March 2014 and declared resolved by the WHO on 10 June 2016. In addition to the devastating effects (more than 11,000 deaths in Guinea, Liberia and Sierra Leone), the Ebola epidemic severely impacted healthcare services and caused setbacks in the treatment and control of HIV, tuberculosis, measles and malaria in these countries.^24^ At that time, there was no vaccine available. The Democratic Republic of the Congo (DRC) is currently grappling with the world’s second largest Ebola epidemic, with more than 2200 deaths and 3400 confirmed infections since the outbreak was declared on 1 August 2018. Vaccination is being implemented to control the current epidemic in DRC.

As far as COVID-19 is concerned, the pangolin, a species threatened with extinction due to poaching for both culinary purposes and traditional Chinese pharmacopoeia, is now suspected of being the “missing link” in the transmission to humans of a virus that probably originated in a species of bat.^25^ Currently, the COVID-19 pandemic is ongoing, and more than 200,000 people have died in 210 countries and territories as of 29 April 2020.

### 6. Vector-borne diseases

West Nile virus (WNV) is a flavivirus that causes West Nile fever. WNV is spread by the bite of a mosquito infected with the virus, and it quickly established itself in North America following its initial recognition in New York City in 1999. Mosquitoes become infected when they feed on infected birds that are natural hosts. WNV has a broad host range (more than 30 mammalian host species). In contrast to the position with birds, it does not spread directly between people. Severe disease may occur in horses (up to 40% mortality).^26^ A vaccine is available for horses.

The plague caused by the bacterium *Yersinia pestis* is infamous as the cause of the Black Death (1347–1353) and the later Second Pandemic (fourteenth to nineteenth centuries CE), when devastating epidemics occurred throughout Europe, the Middle East and North Africa. It has recently been suggested that human ectoparasites, such as human fleas or body lice, were primary vectors for plague during the Second Pandemic, including the Black Death, challenging the assumption that plague was predominantly spread in Europe by rats.^27^ Overall, *Y. pestis* caused three major pandemics, killing 100–200 million individuals overall, making it one of the worst human infectious diseases. Currently, plague epidemics still break out sporadically in various parts of the world, particularly in Madagascar and Africa. Approximately 2000 cases of plague are reported each year to the WHO.

### 7. Prion diseases

Prion diseases or transmissible spongiform encephalopathies (TSEs) are a family of rare, progressive, neurodegenerative disorders that affect both humans and animals. The term “prions” refers to abnormal, pathogenic agents that are transmissible and are capable of inducing abnormal folding of specific normal cellular proteins called prion proteins, which are found most abundantly in the brain. TSEs are distinguished by long incubation periods, characteristic spongiform changes associated with neuronal loss and a failure to induce an inflammatory response. Variant Creutzfeldt–Jakob disease (vCJD) is a prion disease that was first described in 1996 in the UK. There is now strong scientific evidence that the agent responsible for the outbreak of prion disease in cows, bovine spongiform encephalopathy (BSE; or “mad cow” disease), is the same agent responsible for the outbreak of vCJD in humans. In June 2000, the European Union’s (EU) BSE control measures were strengthened by requiring all Member States to remove specified risk materials from animal feed and human food chains. Chronic wasting disease (CWD) is a prion disease that affects deer, elk, reindeer, sika deer and moose. It has been found in some areas of Canada, the USA, Norway and South Korea. To date, there is no strong evidence for any occurrence of CWD in humans. Scrapie is a TSE that affects sheep and goats. It has been known since 1732 and does not appear to be transmissible to humans. There are currently no cures for prion diseases. The unique resistance of prions to classic methods of decontamination and the evidence that prion diseases can be transmitted by medical devices pose a serious infection control challenge to healthcare facilities.^28^

## IV. Disease prevention and control: livestock

### 1. Introductory remarks

Effective prevention and control of transmissible animal diseases, including zoonoses, is a core task of the veterinary services of each OIE Member Country. A number of tools to prevent, control and even eradicate transmissible animal diseases have been developed under the auspices of the OIE and can be accessed online in the Terrestrial Animal Health Code.^29^ The general principles covering the measures described in this section are applicable to multiple diseases.^30^ Importantly, it is emphasised that veterinary services should ensure that any prevention and control programme be proportionate with the risk, be practical and feasible within the national context and be based on a risk analysis.

### 2. Control or eradication of diseases in livestock?

In livestock, the epidemiological unit is a group of animals (not individuals) exposed to the same risk. In the developed world, this usually comprises a farm or production unit (pig and poultry production units, feedlots), as well as live animal markets, exhibitions and trade fairs. Traded animals should also be quarantined before entering a new production unit. Livestock disease programmes are implemented in a given country or region (eg the EU) by the competent national authorities (veterinary services).

Controlling a disease means implementing measures with a view to mitigating the burden of the disease by reducing its prevalence (ie the proportion of a disease that is present in a particular animal population at any given time). This is usually achieved by testing (a proportion) of animals for serological traces of an infection (antibody detection), compulsory vaccinations combined with animal movement restrictions and the implementation of a test-and-slaughter policy (ie the culling of all susceptible animals in highly infected production units). Progress in the programme is measured by the reduction of the incidence of the infection (ie the proportion of new infections in a given animal population at any given time). In essence, a control programme has to be continued indefinitely.

Eradicating a disease means that the infectious agent has to be eliminated from a given animal population. Eradication can be envisaged when the prevalence has been reduced to a level close to 2% of production units. At that point in time, other measures have to come into force. They usually involve a prohibition of vaccination, testing and the implementation of a strict test-and-slaughter policy. This is very costly, but once eradication is achieved (a country is deemed “free from the disease”), there is no more disease burden, and post-eradication measures consist mainly in surveillance measures. In addition, live animals can only be traded internationally if they originate from a country that is free from the disease.

Significantly, some livestock pathogens, such as ASF, survive for a long time in fresh or partially cooked meat or in milk (some pathogens are resistant to pasteurisation). They can also be spread by fomites (ie contaminated inanimate objects, such as clothing). Measures such as banning the importation of animal products are implemented to avoid the reintroduction of a pathogen into a naive population (ie a population in which “herd immunity” towards a given pathogen could not be established because it has been eliminated from the population).

### 3. Vaccines as prevention, control and eradication tools

In 2011, the OIE declared rinderpest (caused by the rinderpest virus, a morbillivirus closely related to the measles and canine distemper viruses) to have been eradicated both from livestock and even-toed ungulate species such as cattle, buffalo, antelope and warthogs. This effort is considered to be one of veterinary medicine’s greatest achievements. On another note, in 1980, the WHO confirmed the global eradication of smallpox (caused by the *Variola major* and *Variola minor* orthopoxviruses) amongst humans.

The eradication of both diseases could not have been achieved worldwide without the extensive use of safe and efficient vaccines.

Veterinary vaccines have had, and continue to have, a major role in protecting animal health and public health, reducing animal suffering, enabling the efficient production of food animals to feed the burgeoning human population and greatly reducing the need for antibiotics to treat food and companion animals. They ensure safe and efficient food production, the control of zoonotic diseases and the control of emerging and exotic diseases in animals and humans, as well as reducing the need for antibiotics.^31^

An important concept in vaccination is that of “herd immunity”. Herd immunity is the protection offered to a group of animals when a sufficiently high proportion of individual animals have been vaccinated. This reduces the numbers of susceptible individuals in an area, as well as the prevalence of disease. This concept is also important in human epidemiology. Unfortunate examples of what can happen when herd immunity diminishes have been the outbreaks of measles in the UK since 2017, which are thought to be due to reduced numbers of children being vaccinated, particularly in Roma communities.^32^

## V. Regulatory frameworks for pandemics of animal origin

So far, no specific international instruments have been adopted with a view to preventing the spread of pandemics of animal origin. That said, the World Trade Organization (WTO) Agreement on the Application of Sanitary and Phytosanitary Measures (SPS Agreement) has enhanced the standards laid down by the OIE, which play a key role in preventing the dissemination of pathogens (Subsection 1). Accordingly, a health approach is intertwined with trade considerations. Secondly, more traditional nature conservation instruments can also contribute to reducing these risks (Subsection 2).

### 1. Trade law as a means for preventing the spread of infectious agents

In the current trend of globalisation, animal health measures have increasing importance to facilitate safe international trade of animals and animal products while avoiding unnecessary impediments to trade. In light of this, the SPS Agreement plays a pivotal role. The focus is placed here on the role of the OIE under the WTO SPS Agreement.

The SPS Agreement elaborates specific rules “for the application of Article XX(b)” of the General Agreement on Tariffs and Trade (GATT) that allows national measures “to protect human, animal and plant life or health”.^33^ In particular, this agreement strikes a delicate balance between the right of the Members to adopt and to maintain measures “necessary to protect human, animal or plant life or health” and the need to restrict the use of such measures for protectionist purposes. Given that SPS measures must necessarily achieve their goals, less trade-restrictive alternatives must be excluded (necessity test).

In virtue of Article 2.2, Members have the right to enact SPS measures inasmuch as they are based upon “scientific principles” and are not maintained without “sufficient scientific evidence”. Furthermore, pursuant to Article 2.3, SPS measures may not be chosen arbitrarily or give rise to “unjustifiable restriction or disguised restriction on trade”.

In accordance with Article 3.1, WTO Members shall base their sanitary or phytosanitary measures on international standards, guidelines or recommendations and may choose measures that “conform to international standards” (eg OIE). The SPS measures that conform to these international standards shall be deemed to be necessary to protect human, animal or plant life or health and presumed to be consistent with the relevant provisions of this Agreement and of GATT 1994.

Nonetheless, Article 3.3 allows WTO Members to introduce or maintain a distinctively higher level of protection than these international standards, ins far as their measures are:

- Scientifically justified; or
- Adopted “as a consequence of the level of sanitary or phytosanitary protection a Member determines to be appropriate in accordance with the relevant provisions of paragraphs 1 through 8 of Article 5”.

Where there are no OIE recommendations or if the country chooses a level of protection requiring measures more stringent than the standards of the OIE, these should be based on an import risk analysis conducted in accordance with Chapter 2.1.

Accordingly, the SPS Agreement enhances the role played by the guidelines of the OIE. The OIE has thus become the WTO reference organisation for standard setting for animal health and zoonotic diseases. Pursuant to 3(4) SPS, “Members shall play a full part … in the relevant international organizations and their subsidiary bodies, in particular … the International Office of Epizootics”.

Specific standards and recommendations (ie the Terrestrial Code) have been adopted by the OIE Terrestrial Animal Health Standards Commission (the Code Commission). This Commission draws on the expertise of internationally renowned specialists when adopting international standards. The OIE publishes two codes (Terrestrial and Aquatic) and two manuals (Terrestrial and Aquatic) as the principle reference for WTO members,^34^ which form a key part of the WTO legal framework for international trade. WTO Members should thus align their import requirements with the recommendations contained in the relevant standards of the Terrestrial Code.

In order to enable free access to world animal health data, the OIE provides Internet users with a variety of computer tools designed to fulfil specific user needs. This portal provides easy access to these tools in order to ensure more accurate results while searching for world animal health information.

The World Animal Health Information System, better known as WAHIS,^35^ is an Internet-based computer system that processes data on animal diseases in real time and then informs the international community. In addition, WAHIS–Wild is a system that gathers and presents information on wildlife diseases that are not included in the OIE list, but are considered to require surveillance. These diseases are not subject to compulsory notification, although Member Countries are encouraged to provide information concerning them voluntarily if they are of significant interest for knowledge in the area of animal health.^36^

### 2. Wildlife conservation as a means for preventing the spread of pathogens

In the aftermath of the lockdown of Hubei province, the Chinese Government imposed a total ban on the trade in and consumption of wild animals in order to reduce the risk of virus transfer from animals to human beings. The heart of the matter is thus whether the legal instruments intended to ensure biodiversity conservation are sufficient in addressing the threats stemming from the viruses harboured by wild animals. Although the COVID-19 crisis has highlighted how wild animals can act as a reservoir for hazardous pathogens, the CBD as well as other international nature conservation instruments are addressing wildlife diseases as a threat to biodiversity, not as reservoir of pathogens for livestock and humans. That said, recent research shows that the causes of wildlife population declines have also facilitated the transmission of animal viruses to humans.^37^

In the following sections, we attempt to assess whether wildlife law and food safety law governing the trade in endangered species, bushmeat and trophies provide effective protection against the spread of pathogens.

#### a. The regulation of the trade in endangered animal species

Although the trade in wild animals dates back centuries, it has increased dramatically with the globalisation of the economy and the rise of wealthy consumers eager to acquire exotic wildlife products. As a result, the legal trade has become significant as well as very lucrative. The main problem is that much of the trade is unlawful. As it is highly lucrative, wildlife trafficking has become one of the world’s most profitable organised crime offences and is ranked alongside human trafficking, arms trafficking and drug dealing in terms of profits. Its exact scale is difficult to quantify, although different sources estimate the profits from such trafficking to be between €8 and €20 billion annually.^38^ Wildlife trafficking not only has a devastating impact on biodiversity, threatening to eradicate some species, but is also a vehicle for the spread of pathogens.

On 3 March 1973, the Convention on International Trade in Endangered Species of Wild Fauna and Flora (CITES) was concluded in Washington, DC. The Convention has 183 parties, including the EU.^39^

We shall briefly comment on the three-pronged approach endorsed by CITES. Trade in species “threatened with extinction”^40^ that are listed in Appendix I is prohibited, with some exceptions. Conversely, trade in “species which are not necessarily now threatened with extinction” but “may become so unless trade in specimens of such species is subject to strict regulation in order to avoid utilization incompatible with their survival”^41^ is permitted inasmuch as it is subject to control. These species are listed in Appendix II, which also includes so-called “lookalike species” (ie species whose specimens in trade look like those of species listed for conservation reasons).^42^ The exporting state must authorise the trade by the granting of an export permit based on proper documentation demonstrating the absence of risk. However, the importing states are not required to issue import permits.^43^ Appendix III contains species that are protected in at least one country, which has asked other CITES Parties for assistance in controlling the trade.

The Conference of the Parties is regularly adding to or removing species from Appendix I and II, or moved between them.^44^

Europe is currently a destination market and a hub for trafficking in transit to other regions.^45^ Due to the single market and the absence of systematic border controls within the EU, CITES was implemented uniformly in all EU Member States with the adoption of harmonised legal acts. Accordingly, the obligations laid down in CITES are fleshed out by specific arrangements adopted in the form of Council Regulation (EC) No 338/97 (the Basic Regulation)^46^ and Commission Regulation (EC) No 865/2006 (the Implementing Regulation).^47^ The latter implements most of the currently applicable recommendations of the Conference of the Parties on the interpretation and implementation of CITES provisions.

In order to achieve a high level of environmental protection, the EU has adopted stricter domestic measures than those provided for under CITES. For instance, the Basic Regulation’s prohibition list contained in Annex A covers more species than CITES on the grounds that it encompasses some CITES Appendix II and III species. By the same token, Annex B, which regulates the trade in CITES Appendix II species, also encompasses some CITES Appendix III species.

Although these two Regulations are directly applicable in all EU Member States, they only vaguely specify the enforcement provisions (investigation, prosecution, penalties, etc.).^48^ As a result, Member States must provide for complementary provisions in order to ensure that breaches are punished in an appropriate manner. On 26 February 2016, the European Commission adopted the Communication on an EU Action Plan against Wildlife Trafficking,^49^ which sets out a comprehensive blueprint for joined-up efforts to fight wildlife crime inside the EU and for strengthening the EU’s role in the global fight against these illegal activities.

Many legal scholars consider CITES as “one of the most effective regulatory schemes”^50^ or as “the most successful of all international treaties concerned with the conservation of wildlife”.^51^ That said, CITES does not appear to be an effective legal instrument for dealing with the health risks stemming from the trade in species. Indeed, the scope of CITES encompasses exclusively species that are threatened by international trade. Accordingly, the Convention does cover either species subject to internal trade or species threatened by habitat loss. Of particular salience in this respect is the number of mammal species, one of the reservoirs of pathogens, covered by CITES. Among the 6495 species of currently recognised mammals,^52^ Appendix I lists 318 species, whilst Appendix II lists 513 species.

In addition, CITES is poorly enforced in many countries. The pangolins are a case in point in this regard. There are eight species of pangolin, four of which occur in Asia and with four native to Africa. Pangolins are the world’s most trafficked mammal, and an estimated 1 million pangolins have been poached during the past decade alone. Over recent decades, poachers have been decimating their populations in order to supply meat and scales to domestic and international markets in East and South-East Asia.^53^ The meat is a delicacy, and different parts of the animals are used in traditional medicine such as aphrodisiacs. The consumption of pangolin meat and products enhances the social status of their consumers. Over the years, demand for pangolins has continued to grow sharply as the populations decline.

With the depletion of Asian pangolin populations, the illicit trade has been progressively shifting to Africa. Today, this illegal trade thus involves all eight species. Given the sheer pressure of trade on their survival, pangolins have a long history in CITES. In 1975, several Asian species were already listed in Appendix II.^54^ Other species were subsequently added to that Appendix. At the 17th meeting of the Conference of the Parties to CITES (CoP17, Johannesburg, 2016), all eight species of pangolin were transferred from CITES Appendix II to Appendix I. This change resulted in a prohibition on trade in the eight species of pangolin. In spite of this regulatory improvement, the trade did not peter out. Recently, Vietnam was considered a major hub for pangolin trafficking into China.^55^

The fate of the pangolins clearly illustrates that wildlife trafficking has received a low enforcement priority compared with other forms of trafficking.^56^ Lately, the UN General Assembly has been encouraging the Member States to take decisive steps at the national level to prevent, combat and eradicate the illegal trade in wildlife, on both the supply and demand sides, including by strengthening the legislation necessary for the prevention, investigation and prosecution of such illegal trade, as well as by strengthening enforcement and criminal justice responses.^57^ Along these lines, the fight against wildlife trafficking has also been proclaimed as one of the Sustainable Development Goals agreed upon by heads of state at a UN summit in September 2015.^58^

In spite of these positive recent developments, the various regulations governing trading in wildlife appear to be insufficient to minimise the risks stemming from transmissible animal diseases, including zoonoses.

#### b. The regulation of the trade in bushmeat

Bushmeat is the everyday term for meat from wildlife species that are hunted for human consumption in Africa, Asia and Central or South America. Bushmeat can be smoked, dried or salted, although these processes do not render it non-infectious. Animal species hunted as bushmeat include primates, herbivores (duikers), rodents, reptiles (crocodiles), etc. A recent study in Nigeria showed that bushmeat consumption, especially of rodents, is uniquely related to improved food security. Reliance on a wider diversity of species in food-insecure households may in turn affect their nutrition and exposure to reservoirs of zoonotic infections, whilst also impacting on wildlife conservation.^59^

Alongside habitat destruction, bushmeat hunting is one of the major threats to wildlife conservation. “While bushmeat hunting is predominantly a poor man’s activity, eating wild meat is not”.^60^ Bushmeat is not only extracted for personal consumption, but is also part of a lucrative organised trade, with high prices indicating luxury status.^61^ Lately, many wilderness areas have been opened up to the markets thanks to improved access and faster transportation. Within many developing countries, this has led to a booming trade, which has exacerbated pressure on the hunted species. In effect, the expansion of illegal international trade is likely to heighten the over-exploitation of source populations, which have already been significantly depleted for domestic needs. Moreover, many primate species as well as all pangolin species are falling within the scope of the Appendix I CITES Convention. Other species, such as the blue duiker, are listed in Appendix II.

Bushmeat can be a reservoir for zoonotic agents. In particular, primates pose a higher risk of transmission of zoonotic pathogens to humans given their physiological similarities.^62^ In Africa, human infections with Ebola have been associated with processing meat from infected primates.

Given that illegal imports of meat can present substantial risks to public and animal health, concerns have been raised regarding the illegal import of bushmeat from developing countries into Europe, particularly in terms of the health risks posed to humans and livestock.

Several European countries have reported considerable quantities of meat being imported on commercial passenger flights. In Switzerland, the total annual inflow of illegal meat imports per year was estimated at 1013 tonnes (95% confidence interval: 226–4192) for meat and 8.6 tonnes (95% confidence interval: 0.8–68.8) for bushmeat.^63^ Between 3 and 20 June 2008, 29 Air France flights arriving at Roissy-Charles de Gaulle airport from Central and West Africa were checked between 05:00 and 11:00 hours, when most African flights land. Passengers carrying iceboxes were targeted for inspection. A total of 134 passengers arriving on 29 flights from 14 West and Central African countries were searched, and almost half of them were found to be carrying meat or fish. In total, it was estimated that 63.2 tonnes of meat and fish were imported per week on the Air France routes checked, of which 8% (5.25 tonnes) was bushmeat. Overall, 39% of the bushmeat carcasses were CITES-listed species (Appendix 1: trade banned; Appendix 2: trade restricted).^64^

The import of uncertified meat is illegal or restricted for sanitary reasons. As far as the EU is concerned, the import of uncertified meat or of products from third countries continues to represent an “unacceptable animal health risk”.^65^ According to Commission Regulation (EC) No 745/2004,^66^ all meat and meat products introduced into the EU by travellers are subject to veterinary checks in accordance with Directive 2002/99/EC.^67^ They must be imported from countries from which exports of specified products of animal origin are permitted,^68^ and they must be presented to a border inspection post accompanied by specific documentation.^69^

However, it is difficult for customs authorities to detect the presence of this bushmeat, particularly when it is imported in small quantities. The lack of effective tools for detecting illegal meat imports is limiting the effectiveness of law enforcement efforts. To make matters worse, there is little research regarding the scale of this trade and the amount of bushmeat imported. Some studies have highlighted the emergence of a luxury market for African bushmeat in Europe.^70^

#### c. The regulation of trophy hunting imports

Recently, an article published in the prestigious journal *Science* claimed that trophy hunting bans imperil biodiversity.^71^ This claim has generated considerable debate and controversy, ranging from assertions of inaccuracy to outrage. A consensus view is that trophy hunting is neither the main threat to nor the main opportunity for wildlife conservation, and that a broader debate should be encouraged. So far, trophy hunting has been regulated for the purpose of nature conservation and not for pathogen transmission to livestock and humans.

## VI. Conclusion

Emerging infectious diseases are dominated by zoonoses originating mainly in wildlife. They represent a significant burden on global economies and public health, in particular when these diseases become pandemics. Their emergence is largely driven by socio-economic, environmental and ecological factors.^72^

If we are to make progress in conserving biodiversity and in preventing the emergence of diseases at the wildlife, livestock and human interface, of importance is to understand the role of pathogens in natural populations and, more importantly, how pathogens interact with other drivers of extinction to cause species loss and transmission of diseases.

Currently, there is no vaccine against COVID-19. There is a hot debate on whether a sufficient number of people will have developed immunity after having been exposed naturally to SARS-CoV-2 in order to lead to herd immunity, so that some confinement measures can be lifted before a vaccine becomes available. Importantly, Dr Anthony Fauci stated on 24 April 2020: “We really can’t depend on herd immunity until we get either enough people infected, or enough people vaccinated”.^73^

With the explosion of the COVID-19 pandemic, the world appears to have changed abruptly. There is a distinct before and after. Political responsibilities are having to be shouldered. Solidarity is rising up from the ashes. Businesses are adapting to the new constraints and suddenly discovering the virtues of a future circular economy. The financial sector is being forced to rethink its models. Will this unsettling strangeness end up plunging our contemporary societies into an unprecedented state of vulnerability? And could one say the same about climate change?

Some might think that these questions will fade into the background once the pandemic has been brought under control and we all return to business as usual. But it is this in particular that we have to fear. Numerous scientists and researchers are cooperating on a massive scale at universities and research institutes around the world. This health crisis must make us stop and think about how we deal with risks, how we assess them and even how we talk about them. We need to move to a radically different social model for dealing with risk.

Over the centuries, pandemics have laid down markers between different eras of human society. We must not wait for this new health crisis to be resolved before thinking about a radically new society. It has been suggested that we have entered the “Anthropocene”, a new geological epoch dating from the start of significant human impacts on Earth’s geology and ecosystems, including, but not limited to, anthropogenic climate change.^74^ Worldwide, humankind is facing a new challenge: developing truly transdisciplinary research that encompasses all aspects of risk assessment, analysis and communication related to the prevention, surveillance and responses to emerging and re-emerging pathogens in a sustainable environment.

We have written this article in an attempt to build bridges between disciplines and to raise awareness and mutual understanding concerning the challenges posed to society by emerging diseases.

We must now define a new integrated understanding of the multilevel bio-social pathways that link up society, biology, health and socio-economic attainment in order to achieve the overarching objective of the UN Sustainable Development Goals: peace and prosperity for people and the planet, now and into the future.

## Footnotes

1 Johns Hopkins Corona Resource Center <https://coronavirus.jhu.edu/map.html>.

2 ASF outbreaks have occurred in recent years in Eastern Europe and Russia, with wild boar populations providing the primary vector of transmission. Recently, ASF has been introduced into the wild boar population of the Province of Luxemburg in Belgium. Whole-genome analysis of ASF virus from Belgium showed high overall identity to recent ASF virus strains from Eastern Europe and China.

3 W Rippley et al, “World Scientists’ Warning to Humanity: A Second Notice” (2017) 67(12) BioScience 1026.

4 R Leakey and R Lewin, *The Sixth Extinction: Patterns of Life and the Future of Humankind* (New York, Doubleday 1995). However, it has been calculated that more species went extinct between the times of mass extinctions than during the mass extinctions.

5 Rippley et al, supra, note 3, 1028.

6 Pursuant to the fifth target, by 2020, the rate of loss of all natural habitats, including forests, is at least halved and, where feasible, brought close to zero, and degradation and fragmentation are significantly reduced. As far as the EU is concerned, see Communication from the Commission, Our life insurance, our natural capital: an EU biodiversity strategy to 2020 (COM/2011/0244 final).

7 See Art 6 CDB. Obligations related to these two concepts are further elaborated in Arts 7–14.

8 Art 2. Sustainable use is addressed in Art 10, which requires Parties to adopt measures relating to the use of biodiversity in order to avoid or minimise impacts on biological diversity.

9 See Secretariat of the Convention on Biological Diversity, *Addis Ababa Principles and Guidelines for Sustainable Use* (Montreal, Secretariat of the Convention on Biological Diversity 2004).

10 NG Yoccoz et al, “Biodiversity May Wax or Wane Depending on Metrics or Taxa” (2018) 115(8) Proceedings of the National Academy of Sciences of the United States of America 1681.

11 KF Smith et al, “The Role of Infectious Diseases in Biological Conservation” (2009) 12(1) Animal Conservation 1.

12 OIE <https://www.oie.int>.

13 FAO <http://www.fao.org/home/en>.

14 IP Chatziprodromidou et al, “Global Avian Influenza Outbreaks 2010–2016: A Systematic Review of Their Distribution, Avian Species and Virus Subtype” (2018) 7(1) Systematic Reviews 17.

15 ME Nickol et al, “A Year of Terror and a Century of Reflection: Perspectives on the Great Influenza Pandemic of 1918–1919” (2019) 19(1) BMC Infectious Diseases 117.

16 Centers for Disease Control and Prevention <https://www.cdc.gov/flu/swineflu/variant/h3n2v-cases.htm?CDC_AA_refVal=https%3A%2F%2Fwww.cdc.gov%2Fflu%2Fswineflu%2Fh3n2v-cases.htm>.

17 WHO <https://www.who.int/influenza/pip/en>.

18 D Haydon et al, “The UK Foot-and-Mouth Disease Outbreak – The Aftermath” (2004) 2 Nature Reviews. Microbiology 675.

19 F Jori et al, “Transmission of Foot and Mouth Disease at the Wildlife/Livestock Interface of the Kruger National Park, South Africa: Can the Risk Be Mitigated?’ (2016) 12 Preventive Veterinary Medicine 19.

20 D Venkatesh et al, “Avian Influenza Viruses in Wild Birds: Virus Evolution in a Multihost Ecosystem” (2018) 92(15) Journal of Virology e00433-18.

21 LM Looi et al, “Lessons from the Nipah Virus Outbreak in Malaysia” (2007) 29(2) Malaysian Journal of Pathology 63.

22 SP Luby et al, “Recurrent Zoonotic Transmission of Nipah Virus into Humans, Bangladesh, 2001–2007” (2009) 15(8) Emerging Infectious Diseases 1229.

23 HM De Nys et al, “Survey of Ebola Viruses in Frugivorous and Insectivorous Bats in Guinea, Cameroon, and the Democratic Republic of the Congo, 2015–2017” (2018) 24(12) Emerging Infectious Diseases 2228.

24 AS Parpia et al, “Effects of Response to 2014–2015 Ebola Outbreak on Deaths from Malaria, HIV/AIDS, and Tuberculosis, West Africa” (2016) 22(3) Emerging Infectious Diseases 433.

25 T Zhang et al, “Probable Pangolin Origin of SARS-CoV-2 Associated with the COVID-19 Outbreak” (2020) 30(7) Current Biology 1346.

26 AT Ciota, “West Nile Virus and Its Vectors” (2017) 22 Current Opinion in Insect Science 28.

27 K Dean et al, “Human Ectoparasites and the Spread of Plague in Europe during the Second Pandemic” (2018) 115(6) Proceedings of the National Academy of Sciences of the United States of America 1304.

28 T Cassandra et al, “Recent Advances in Understanding Mammalian Prion Structure: A Mini Review’ (2019) 12 Frontiers in Molecular Neuroscience 169.

29 <https://www.oie.int/en/standard-setting/terrestrial-code/access-online>.

30 <https://www.oie.int/index.php?id=169&L=0&htmfile=titre_1.4.htm>.

31 J Roth, “Veterinary Vaccines and Their Importance to Animal Health and Public Health” (2011) 5 Procedia in Vaccinology 127.

32 S Bell et al, “What Have We Learnt from Measles Outbreaks in 3 English Cities? A Qualitative Exploration of Factors Influencing Vaccination Uptake in Romanian and Roma Romanian Communities” (2020) 20(1) BMC Public Health 381.

33 Preamble, last sentence.

34 <https://www.oie.int/en/international-standard-setting/overview>.

35 <https://www.oie.int/wahis_2/public/wahid.php/Wahidhome/Home/index/newlang/en>.

36 <https://www.oie.int/wahis_2/public/wahidwild.php/Index/indexcontent/newlang/en>.

37 CK Johnson et al, “Global Shifts in Mammalian Population Trends Reveal Key Predictors of Virus Spillover Risk” (2020) 287(1924) Proceedings of the Royal Society B 20192736.

38 See the UN Office on Drugs and Crime Global Programme for Combating Wildlife and Forest Crime.

39 Council Decision (EU) 2015/451 of 6 March 2015 concerning the accession of the European Union to the Convention on International Trade in Endangered Species of Wild Fauna and Flora (CITES), OJ L 75, 19 March 2015, p 1.

40 Art II(1).

41 Art II(2).

42 Art II(2).

43 Although a permit is needed in some countries that have taken stricter measures than CITES requires.

44 Art XV.

45 The European Commission adopted Communication on an EU Action Plan against Wildlife Trafficking, § 1.

46 Council Regulation (EC) No 338/97 of 9 December 1996 on the protection of species of wild fauna and flora by regulating trade therein, OJ L 061, 3.3.1997, p 1.

47 Commission Regulation (EC) No 865/2006 of 4 May 2006 laying down detailed rules concerning the implementation of Council Regulation (EC) No 338/97 on the protection of species of wild fauna and flora by regulating trade therein, OJ L 166, 19 June 2006, p 1.

48 Basic Regulation, Art 16. In addition, Directive 2008/99/EC of 19 November 2008 on the protection of the environment through criminal law requires Member States to adopt dissuasive penalties for trading in specimens of protected wild fauna or flora species or parts or derivatives thereof.

49 COM/2016/087 final.

50 P Birnie, A Boyle and C Redgwell, *International Law and the Environment* (Oxford, 3^rd^ edn, Oxford University Press 2002) p 685.

51 M Bowman, P Davies and C Redgwell, *Lyster’s International Wildlife Law* (Cambridge, 2^nd^ edn, Cambridge University Press 2010) p 484.

52 CJ Burgin, JP Colella, PL Kahn and N Upham, “How Many Species of Mammals Are There?” (2018) 99(1) Journal of Mammalogy 1.

53 Environmental Investigation Agency, *Running Out of Time: Wildlife Crime Justice Failure in Vietnam* <https://eia-international.org/wp-content/uploads/EIA-report-Running-out-of-Time.pdf> 10.

54 IUCN, Report for the CITES Secretariat of D. Challender and C. Waterman, “Implementation of CITES Decisions 17.239 b) and 17.240 on Pangolins (*Manis* spp.)”, September 2017, 11.

55 Environmental Investigation Agency, supra, note 51, 10.

56 The International Consortium on Combating Wildlife Crime, a collaborative effort of the secretariat of CITES, INTERPOL, the UN Office on Drugs and Crime, the World Bank and the World Custom Organization, provides technical assistance to UN Member States.

57 UN General Assembly, Resolution 69/314 of 30 July 2015, Tackling illicit trafficking in wildlife, § 3.

58 Goal 15 of the 2030 Agenda for Sustainable Development sets the target of “tak[ing] urgent action to end poaching and trafficking of protected species of flora and fauna, and address[ing] both demand and supply of illegal wildlife products” (target 15.7).

59 S Friant et al, “Eating Bushmeat Improves Food Security in a Biodiversity and Infectious Disease ‘Hotspot’” (2020) 17(1) Ecohealth 125.

60 LifeWatch Belgium, “Identifying African bushmeat for Sale in Brussels Using DNA” <http://www.lifewatch.be/en/identifying-african-bushmeat-sale-brussels-using-dna>.

61 A-L Chaber et al, “The Scale of Illegal Meat Importation from Africa to Europe via Paris” (2010) 3(5) Conservation Letters 317.

62 H Falk et al, “Illegal Import of Bushmeat and of Meat Products into Switzerland on Commercial Passenger Flights” (2013) 32(3) Revue scientifique et technique (International Office of Epizootics) 727.

63
ibid.

64 Chaber et al, supra, note 59.

65 Preamble, no. 9.

66 Art 1(2) of Commission Regulation (EC) No 745/2004 of 16 April 2004 laying down measures with regard to imports of products of animal origin for personal consumption, OJ L 122, 26 April 2004, p 1.

67 Council Directive 2002/99/EC of 16 December 2002 laying down the animal health rules governing the production, processing, distribution and introduction of products of animal origin for human consumption, OJ L 18, 23 January 2003, p 1.

68 Council Directive 2002/99/EC, Art 8.

69
ibid, Art 9.

70 A-L Chaber et al, supra, note 59.

71 A Dickman, “Trophy Hunting Bans Imperil Biodiversity” (2019) 6456 Science 874.

72 K Jones et al, “Global Trends in Emerging Infectious Disease” (2008) 451(7181) Nature 990.

73 <https://time.com/5825386/herd-immunity-coronavirus-covid-19>. Dr Anthony Fauci is the director of the National Institute of Allergy and Infectious Diseases and a member of the White House Coronavirus Task Force.

74 <https://en.wikipedia.org/wiki/Anthropocene>.

